# Gene expression profile analysis of tobacco leaf trichomes

**DOI:** 10.1186/1471-2229-11-76

**Published:** 2011-05-08

**Authors:** Hong Cui, Song-Tao Zhang, Hui-Juan Yang, Hao Ji, Xiu-Jie Wang

**Affiliations:** 1 Key Laboratory for Cultivation of Tobacco Industry, College of Tobacco Science, Henan, Agricultural University, Zhengzhou, 450002, P. R. China; 2 State Key Laboratory of Plant Genomics, Institute of Genetics and Developmental Biology, the Chinese Academy of Sciences, Beijing 100101, P. R. China

## Abstract

**Background:**

Leaf trichomes of *Nicotiana tabacum *are distinguished by their large size, high density, and superior secretion ability. They contribute to plant defense response against biotic and abiotic stress, and also influence leaf aroma and smoke flavor. However, there is limited genomic information about trichomes of this non-model plant species.

**Results:**

We have characterized *Nicotiana tabacum *leaf trichome gene expression using two approaches. In the first, a trichome cDNA library was randomly sequenced, and 2831 unique genes were obtained. The most highly abundant transcript was ribulose bisphosphate carboxylase (RuBisCO). Among the related sequences, most encoded enzymes involved in primary metabolism. Secondary metabolism related genes, such as isoprenoid and flavonoid biosynthesis-related, were also identified. In the second approach, a cDNA microarray prepared from these 2831 clones was used to compare gene expression levels in trichome and leaf. There were 438 differentially expressed genes between trichome and leaves-minus-trichomes. Of these, 207 highly expressed genes in tobacco trichomes were enriched in second metabolic processes, defense responses, and the metabolism regulation categories. The expression of selected unigenes was confirmed by semi-quantitative RT-PCR analysis, some of which were specifically expressed in trichomes.

**Conclusion:**

The expression feature of leaf trichomes in *Nicotiana tabacum *indicates their metabolic activity and potential importance in stress resistance. Sequences predominantly expressed in trichomes will facilitate gene-mining and metabolism control of plant trichome.

## Background

Many terrestrial plants are covered with uni- or multi-cellular epidermal appendages called trichomes. Plant trichomes frequently function as the first line of defense against biotic and abiotic stresses by space hindrance [1]. Some plant species bear glandular trichomes that secrete a series of lipophilic substances and proteins, and are distinguished for their medicinal, culinary, fragrant and insecticidal properties. Functional genomic approaches are now emerging as powerful tools that can accelerate our understanding of trichomes. Significant progress has been made in cell differentiation and development research, particularly in *Arabidopsis thaliana *[2] and cotton [3]. However, limited information about metabolism and secretion can be obtained from these model plants as non-glandular trichome species, whereas several plant species can be more attractive in trichome metabolism research. *Mentha piperita *glandular trichomes are specialized structures for monoterpene synthesis, which are the major compounds of and give the characteristic flavor to mint oil. Its cDNA library has been sequenced, and candidate genes putatively involved in essential oil metabolism were cloned and transformed for the purpose of genetic engineering of essential oil biosynthesis [4]. *Artemisia annual *glandular trichomes synthesize and secrete the most important anti-malarial compound, artemisinin, an endoperoxide sesquiterpene lactone. Its glandular trichome plasmid cDNA library was established and randomly sequenced as starting material for dissecting isoprenoid biosynthesis [5]. Furthermore, trichome gene expression profile analysis of other plant species, such as sweet basil [6], alfalfa [7], and hop [8], has also been studied. According to the results, the characteristics of trichome gene expression differ in plant species, being closely related to morphology, structure, development and metabolism features.

Tobacco trichomes are distinguished by their large size, high density, and superior secretory ability. They cover the entire plant throughout the whole development stage, and make the plant very sticky. There are two main types of glandular trichomes on tobacco leaves, short trichomes with a unicellular stalk and a multicellular head, and tall trichomes with a multicellular stalk possessing uni- or multi-cellular heads. Cembrenoid diterpenes are one of the most important components of the exudates, which have wide-ranging biological activities including insect trail pheromones, neurotoxins, cytotoxins, anti-inflammatory and antimitotic activity [9]. In addition to their contribution to plant resistance, a positive effect of trichome exudates on leaf aroma and smoking flavor has also been proved [10]. However, in contrast to the broad knowledge on tobacco trichome morphology and chemistry, much less is known about gene expression of these special structures.

The initiative work on gene-mining from tobacco trichomes was reported in 2001. A trichome-specific P450 hydroxylase gene, *CYP71D16 *was cloned and functionally characterized. **S**uppression of its expression by RNAi changed the profile of the terpenoid spectrum of trichome exudates. Transgenic plants showed enhanced resistance against aphids [11]. More recently, trichome cDNA libraries of control and cadmium-treated plants have been randomly sequenced. Antipathogenic T-phylloplanin-like protein, glutathione peroxidase, and several class of pathogensis-related protein (PR) were expressed predominantly in Cd-treated trichomes, indicating that the tobacco trichome is a metabolic active and stress-responsive organ [12]. Genes expressed in tobacco trichomes during development, metabolism, and their protective function remain mostly unknown. To monitor the gene expression of tobacco trichome on a relatively large scale, we constructed a leaf trichome cDNA library using the species *N. tabacum *L. cv. K326, a widely grown cultivar in China. From over 5000 high quality sequences, we obtained 2831 unique ESTs. Custom-designed cDNA microarrays of these ESTs were used to analyze the gene expression of trichome. By probing the cDNA microarrays with RNA samples from trichomes and leaves-minus-trichomes, 207 upregulated genes in trichomes were identified, and were the foundation for further investigation.

## Results

### Leaf trichomes isolation and ESTs analysis

**Figure 1:**
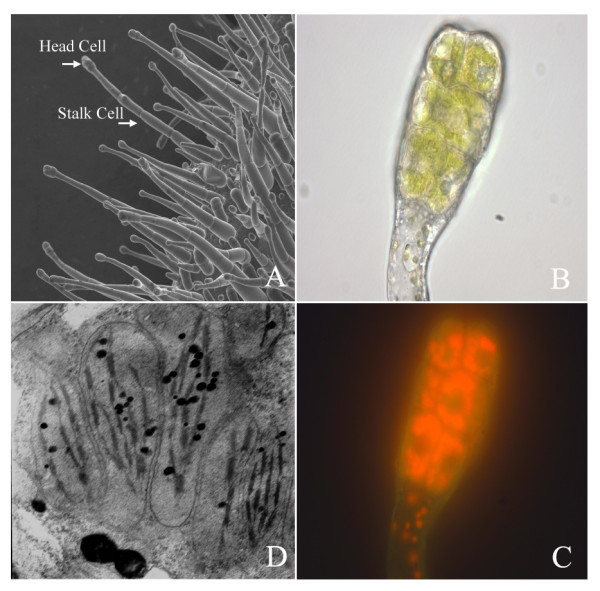
**Cytological examination of tobacco trichomes**. (A) Scanning electron micrograph of tobacco young leaf (~2 cm long) showing trichomes with head cells and stalk cells (× 120). (B) Light micrograph of the mature trichome showing head cells (× 1000). (C) Fluorescence microscopy of the trichome of (B), showing intensive red fluorescence of chlorophyll in the head cells (× 1000). (D) Transmission electron micrograph of trichomes, showing the chloroplast structures in the head cells (× 15,000).

Flourishing one-head-cell trichomes were found on the tobacco leaves surface when they emerged (Figure 1A). When leaves were 40-50 cm long, the structural development was basically completed. Most of the leaf trichomes at this stage were also well developed. There were 8-12 cells in the head of each trichome. Cytoplasm of the head cells was much denser than that of the stalk cells (Figure 1B). Intensive red fluorescence was emitted from the head cells, showing high chlorophyll content (Figure 1-C). Chloroplasts with perfect thylakoid structures and osmiophilic particles were also found in the head cells by ultra structural microscopy (Figure 1-D). These morphology features clearly showed that, at this stage, trichomes were biologically active and ideal for analysis of their gene expression.

A cDNA library was constructed from leaf trichomes. The randomly selected individual clones were sequenced from the 5'-terminus. High-quality sequences of 5139 clones were annotated and clustered into contigs, representing 2831 unique genes. Among them, 2246 genes were singletons, indicating the low redundancy of the constructed library. A total of 585 genes were presented in multiple clones, ranging from low redundancy (2-5 ESTs per contig for 487 contigs) through medium redundancy (6-20 ESTs per contig for 77 contigs) to high redundancy (> 20 ESTs per contig for 21 contigs). The largest contig in the database, showing sequence similarity to RuBisCO, had 133 ESTs (Table 1). This finding is apparently in consistent with the morphology characteristic of trichomes.

**Table 1:** The 20 most abundant ESTs in the tobacco leaf trichome library with gene annotation of their closest hit identified by Blastx

No. of ESTs	Gene ID	Gene annotation of closest hit	*E *value
133	59800169	Ribulose bisphosphate carboxylase small chain (*N. sylvestris*)	E-92
60	115805	Chlorophyll a-b binding protein 40 (*N. tabacum*)	7E-83
55	119583048	RAS and EF-hand domain containing (*Homo sapiens*)	E-30
51	131015	Pathogenesis-related protein *(N. tabacum*)	E-55
42	11558417	Endochitinase (*N. sylvestris*)	2E-71
42	112983654	Bombyrin (*Bombyx mori*)	4E-8
39	3913932	Proteinase inhibitor type-2 precursor (*N. tabacum*)	7E-69
38	110638395	Probable sulphatase (*Cytophaga hutchinsonii*)	9.7
33	45738252	Auxin-repressed protein (*Solanum virginianum*)	4E-34
31	3790355	Chitinase 134 (*N. tabacum*)	6E-91
30	111218904	Ubiquitin (*A. thaliana*)	1E-55
30	23506611	histone H1D (*N. tabacum)*	2E-47
26	90992878	Phylloplanin (*N. tabacum)*	2E-23
26	130826	Pathogenesis-related protein 1A precursor (*N. tabacum*)	9E-88
26	19771	Acidic chitinase PR-P (*N. tabacum*)	2E-38
25	66513545	Hypothetical protein (*Apis mellifera*)	2.6
24	2497901	Metallothionein-like protein type 2 metallothionein	4E-25
23	111218906	Ubiquitin (*A. thaliana*)	3E-76
22	30013665	Chloroplast thiazole biosynthetic protein *(N. tabacum*)	2E-77
21	170337	mRNA inducible by salicylic acid or by TMV Systemic Acquired Resistance response (*N. tabacum*)	4E-30

**Figure 2:**
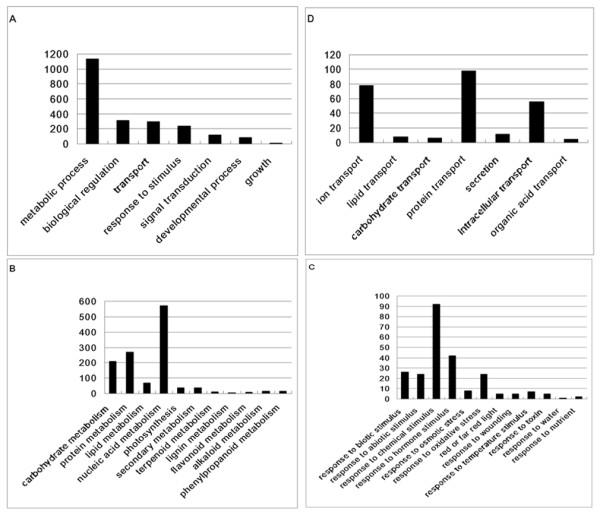
**Function analysis of tobacco leaf trichome ESTs**. (A) GO categories of biological process. (B) GO categories of metabolism. (C) GO categories of stimulus response. (D) GO categories of transport. The results were based on EST counts from a total of 1844 annotated ESTs.

Among the 2,831 unigenes, 34.9% (987) has no reported homologs or showed homology to the genes coding for predicted proteins with unknown function (expect valued < 1.0 E^-5^) as analyzed by the BLAST program against analysis data from the non-redundant protein (NR) database. The high percentage of unidentified genes suggests that tobacco leaf trichome is an interesting source for gene-mining. Other 65.1% (1844) of the unique genes have defined functions (http://amigo.geneontology.org). GO categories of these 1844 annotated genes are given in Figure 2. Under the category of biological process, proteins encoded by 61.5% ESTs were putatively involved in metabolic processes, the largest functional group among our EST database. Other groups were related to biological regulation (16.8%), transport (16.1%), stimulus response (12.9%), signal transduction (6.5%), developmental process (4.4%), and growth (0.6%), respectively (Figure 2A).

Within the metabolic category (Figure 2B), the primary metabolism group (including carbohydrate, protein, nucleic acid, and lipid metabolic process) was predominantly represented. 37 photosynthesis related genes were also cloned, indicating the photosynthetic activity of chloroplasts in tobacco trichomes. Secondary metabolism (including isoprenoid, flavonoid, lignin, alkaloid, and phenylpropanoid metabolic process) accounted for ~3% of total metabolism-related sequences, which seemed much lower than in other plant species. GO categories of stimulus response were shown in Figure 2C. As expected, a significant number of genes related to abiotic stress, such as osmotic, temperature, light, water, wounding, and oxidation. Besides, genes responding to chemicals, such as toxin, nutrient and hormone were also found, suggesting the complexity of biological regulation of tobacco trichomes. Another large group was transport related-genes (Figure 2D). Some secretion related genes and intracellular transport genes were found. Genes representing proteins for the transportation of ion, lipid, carbohydrate, protein and organic acid were also identified, supporting the secretory function of tobacco trichomes.

### Microarray analysis of trichome-expressed genes

The entire set of 2831 trichome cDNAs were amplified and spotted at high density on glass microscope slides (ArrayExpress accession: A-MEXP-2007). To identify the features of genes expression of leaf trichomes, microarray analysis was performed between trichomes and leaves-minus-trichomes. Each glass slide held 3 copies of the entire array. To ensure the reliability of the results, 2 microarray slides (6 replicates) were used for each experiment. Two independent RNA preparations were made for each analysis, and labeling of the cDNA (Cy3 versus Cy5) was reversed on the second slide. RNA extracted from trichomes and leaves-minus-trichomes was used as probes to compare gene expression between the two organs. Their correlation coefficient of the ratios was 0.9, suggesting good reproducibility among individual arrays in the same experiment.

**Figure 3:**
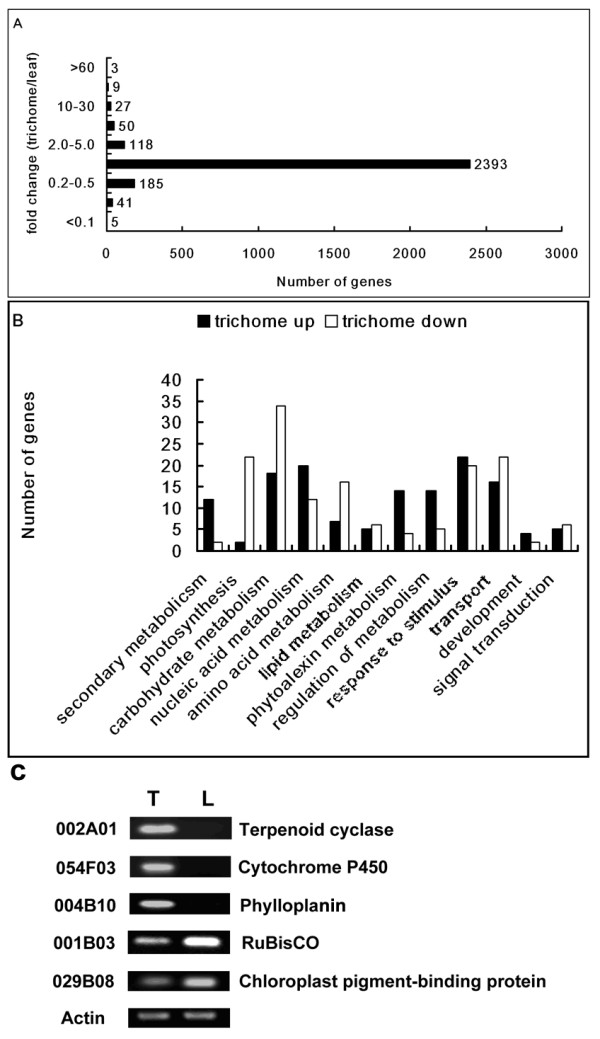
**Detection of genes differentially expressed in tobacco trichomes and leaves by microarray analysis**. A. Distribution of genes in various fold change categories based on the ratio of expression levels of trichomes compared to leaves-minus-trichomes. B. Gene ontology classifications (biological process) for differentially expressed genes between trichomes and leaves. C. Tissue specific expression of selected unigenes. Semi-quantitative RT-PCR was performed using total RNAs from trichomes (T) and leaves-minus-trichomes (L). Terpenoid cyclase (002A01), Cytochrome P450 (054F03) and Phylloplanin (004B10) expressed specifically in trichomes.

After correction for redundancy, the distribution of genes in various fold-change categories based on the ratio of expression of trichomes compared to leaves are shown in Figure 3A. Setting a 2-fold change in gene expression as the threshold, 84.5% genes (2393) had equal expression levels in the trichomes and leaves. 438 differentially expressed genes were identified, of which 207 were expressed more strongly in trichomes (see additional file 1), while the other 231 genes showed lower expression. Most of the high differentially expressed genes were 2.0-5.0 fold increased. There were 12 genes with > 30-fold increased in expression, the highest one increased 67 fold. These genes are worth to be followed in future study.

GO function categories for differentially expressed genes between trichomes and leaves were compared. A total of 63.7% of highly expressed genes and 70.6% of low expressed genes of trichomes were annotated http://amigo.geneontology.org. The predicted gene sets for the high and the low expression were distributed among the biology processes categories (Figure 3B). Most of the differentially expressed genes between trichomes and leaves were metabolism-related. 12 genes encoding enzymes of secondary metabolic process, mainly terpenoid biosynthesis and phenylpropanoid, were highly expressed in trichomes. Only 2 genes related to nicotinamide metabolism were highly expressed in leaves. In contrast, most of the primary metabolism-related genes were expressed much strongly in leaves than in trichomes, especially those relevant to carbohydrate and protein metabolism. Comparatively, RNA and DNA metabolic processes were more active in trichomes. 22 photosynthesis-related genes, including light and dark responsive genes, were clearly elevated in leaves. Highly expressed genes related to phytoalexin and resistant responses were predominantly expressed in trichomes. Although the number of highly expressed genes with biological regulation functions in trichomes and leaves was almost the same, much more metabolic regulation genes were present in trichomes. Very few genes encoding enzymes of development and signal transduction were found in the differentially expressed gene category. Although transport-related genes were more highly expressed in leaves, secretion-related genes were predominant in trichomes.

The 20 most preferentially expressed genes in trichome, according to the microarray data, are shown in Table 2, including the 2 most highly expressed genes, 014D04 [Refseq: NP_001068510] and 003C02 [Refseq: ZP_01980035], of unknown function. There were 5 genes, 002H12 [GenBank: BAF44533], 021F05 [EMBL: CAA55812], 070E09 [Refseq: NP_563842], 002G01 [EMBL: CAN73039] and 022G07 [Swiss-Prot: Q56S59], functionally associated with stimulus responses. Gene 002A01 [GenBank: AAS46038] and 073A12 [Swiss-Prot: P22928] were related to terpenoid biosynthesis and phenylpropanoid biosynthesis, respectively. 057E12 [GenBank: ABI54118] that encoded an enzyme homologous with caffeic acid-methyltransferase was relevant to cell wall metabolism [13]. 033B07 [Refseq: NP_190041] that encoded an acyl-CoA-reductase-like protein was thought to contribute to wax ester biosynthesis [14]. The other genes were related to protein metabolism, 001G11[Swiss-Prot: Q40561], 012G04 [EMBL: CAJ17242], and 013D11[Refseq: XP_572078], carbohydrate metabolism 023D08 [EMBL: CAN77531], and iron binding 059E07 [EMBL: CAN77062].

**Table 2:** The 20 genes with the highest expression level in trichomes determined by microarray transcriptome analysis

**Clone ID**^**a**^	Gene ID	**Gene annotation**^**b**^	**Ratio(T/L)**^**c**^
014D04	154317162	Unknown *[Botryotinia fuckeliana B05.10]*	67.1
003C02	153827368	Unknown *[Vibrio cholerae MZO-2]*	65.3
002H12	121663827	Class IV chitinase *[N. tabacum]*	62.3
023D08	147802595	Hydrolyzing O-glycosyl compounds *[Vitis vinifera]*	56.9
057E12	114199046	Caffeic acid O-methyltransferase [*Malus × domestica]*	55.8
021F05	860903	Sn-1 (defense response) *[Capsicum annuum]*	44.9
022C03	110769331	Serine-type endopeptidase activity *[Apis mellifera]*	42.2
002A01	42795423	Terpenoid cyclase *[N. tabacum]*	41.4
059E07	147837626	Iron ion binding *[Vitis vinifera]*	34.4
070E09	6782438	Glycine-rich protein *[Nicotiana glauca]*	34.1
001G11	3913932	Proteinase inhibitor type-2 precursor *[N. tabacum]*	33.1
012G04	70909635	Ribosomal protein L7Ae *[Curculio glandium]*	30.0
013D11	58269844	40S ribosomal protein S8 *[Cryptococcus neoformans]*	28.8
038H01	111069317	Unknown *[Phaeosphaeria nodorum SN15]*	26.8
033B07	145339118	Acyl CoA reductase -like protein *[A. thaliana]*	24.0
073A12	231805	Chalcone synthase *[Petunia × hybrida]*	23.5
001D10	110638395	Sulphatase *[Cytophaga hutchinsonii]*	23.3
009A09	21700771	Unknown *[Glycine max]*	23.0
002G01	147828182	Response to abscisic acid stimulus *[Vitis vinifera]*	22.5
022G07	68052840	Phylloplanin precursor *[N. tabacum]*	22.1

a: The number of gene clones of tobacco cDNA library.

b: Best blast hit

c: Trichomes/Leaves-minus-trichomes

Five genes involved in terpenoid biosynthesis process, stress responses, and photosynthesis respectively were selected for semi-quantitative RT-PCR analysis to confirm their expression patterns in trichomes and leaves. PCR experiments were conducted on 2 RNA pools derived from trichomes and leaves-minus-trichomes (Figure 3C). The results demonstrated that all the 5 selected genes were clearly expressed in trichomes, 3 were highly expressed and 2 were weakly expressed, consistent with the microarray data. Gene 002A01 [GenBank: AAS46038] (41.4-fold) putatively encoded a protein homolog to tobacco terpenoid cyclase. 054F03 [GenBank: AAD47832] (10.7 fold) was a homolog of the cytochrome P450 gene, *CYP71D16*, involved in diterpenoid biosynthesis of tobacco trichomes [15]. Both the two genes were expressed exclusively in trichomes. No amplified signals were found in leaves- minus-trichomes in the RT-PCR analysis. 004B10 [GenBank: ABE03627] (15.9 fold), putatively encoding T-phylloplanin-like protein, was also expressed specifically in tobacco trichomes. The other 2 selected genes, 001B03 [Swiss-Port: P69249] (0.408-fold), homolog of the RuBisCo small chain, and 029B08 [GenBank: ABG73415] (0.31-fold), homolog of chloroplast pigment-binding protein CP29, were prominently expressed in both leaves and trichomes.

## Discussion

Although *Nicotiana tobacum *may currently lack whole genome information as compared to other model plants, it provides a better platform for elucidating economically important secondary metabolites. Previously, only limited genomic information on tobacco trichome is available in scattered databases. TrichOME http://www.planttrichome.org/trichomedb/ is an integrated genomic database of genes and metabolic pathway in plant trichomes [16]. It currently contains 950,025 ESTs sequenced from 14 species, including *Nicotiana tobacum*. In total 7,112 tobacco unigenes from 4 EST trichome libraries have been displayed. A blastx search against TrichomeOME showed that only 474 (16.8%) of our unigenes had good blast hits (e-value < 1e-5). Thus our cDNA library sequencing of tobacco trichome had many transcripts that had not previously been detected. No microarray analysis of tobacco trichome is available in the public database at present, and the gene expression characteristics of tobacco trichomes seem far from being comprehensively understood. Combining large-scale random sequencing with gene expression analysis has provided a unique and comprehensive overview of transcription related to key metabolic pathways in tobacco trichomes.

### Primary metabolism in tobacco trichomes

Plant trichomes are special organs frequently functioning as plant defense. Secondary metabolism is often supposed to be the most predominant metabolic process in trichomes. Conversely in our analysis, tobacco trichomes were mostly involved in primary metabolic and photosynthetic activities. The largest contig in the tobacco trichome EST library obtained is homologous to RuBisCO, an enzyme involved in the Calvin cycle that catalyzes the first major step in carbon fixation. Unigenes functional ontology analysis showed that genes related to primary metabolism and photosynthesis were among the most abundant categories in tobacco trichomes. Several other reports made a similar discovery. Comparative proteomics showed that RuBisCO was among the spots that were highly enriched in trichomes at the later stage in leave development [17]. Sequencing of tobacco trichomes cDNA library constructed from cadmium-treated leaves also proved that genes for photosynthesis and primary metabolism were detected with high frequency [12]. However, this discovery is quite different from that of other plant species, such as *Mentha piperita*, in which photosynthesis-related genes are totally absent. Secondary metabolism accounts for ~35% of total metabolism in the trichomes ESTs [4]. Morphology and structure observation offer some support for this phenomenon. Peppermint trichomes contain no chloroplasts, but leucoplasts [18], while plenty of developed chloroplasts and apparently red chlorophyll fluorescence were readily observed in the head cells of tobacco trichomes. The structure of chloroplasts and the intensity of chlorophyll fluorescence in tobacco trichomes routinely changed with the leaf development stage [19], and were also affected by environmental factors, such as drought [20] and nutrient allocation [21], implying that trichome chloroplasts are biological active and the regulation mechanism is very complicated. However, the precise role of the chloroplasts in the special glandular organ remains unknown.

We found the RuBisCO gene was relative weakly expressed in trichomes compared with leaves by both microarray and RT-PCR analysis. Some other genes related to photosynthesis were also highly expressed in leaves. It is supposed that, at least, tobacco trichomes partially offer the energy and precursors for secondary substance synthesis and secretion processes by them. Interestingly, the main secretion of peppermint and tobacco trichomes both belong to terpene family (monoterpenoid and diterpenoid, respectively), but their mechanisms of biosynthesis may be totally different.

### Terpene metabolism

Terpenes are the most abundant compounds synthesized in plant trichomes, and certainly the main focus in trichome metabolism research. Volatile monoterpene and sesquiterpene are the main trichome secretions in most plant species. Tobacco trichomes specifically synthesize and secrete diterpene [22], non-volatile cembretriene-diols (CBT-diols) contribute as high as ~60% of trichome exudate weight in *N. tabacum*, T.I. 1068 [11]. Thus tobacco trichomes are an important source of novel diterpene biosynthesis-related genes mining.

A group of genes involved in terpenoid metabolism were annotated during trichome cDNA library sequencing, but fewer than expected. This was probably due to the primary metabolism-related genes being much more abundant and only limited clones were selected. Other reasons may be the particularity of terpenoid metabolism in tobacco trichome, and the relative paucity of sequence information for the *Nicotiana *genus in the public databases. Although these genes accounted for a very low proportion of tobacco trichome ESTs, they all showed dramatically increased expression level compared to leaves. Gene 002A01 [GenBank: AAS46038] homology to a terpenoid cyclase was expressed 41 fold higher in trichomes than in leaves. No target fragment of this gene was amplified in leaves-minus-trichomes by RT-PCR analysis, indicating that it is expressed specifically in trichomes. Since diterpenes are the only kind of terpenoids thought to be specially synthesized in trichomes, clone 002A01 is the most likely one involved in diterpenoid biosynthesis, and awaits further analysis. Clone 054F03 is definitely diterpenoid biosynthesis-related. Its sequence is homology to tobacco cytochrome P450 gene *CYP71D16*, a cembratrieneol cyclase gene responsible for conversion CBT-ols to CBT-diols [15]. This gene also uniquely expressed in trichomes according to both microarray data and RT-PCR amplification. Except for putatively diterpene biosynthesis-related genes, no other genes involved in terpenoid metabolism pathway were found in the trichome up regulated category. It is certain that diterpene metabolism occurs predominately and specifically in tobacco trichomes. Recently several reports have focused on the cytosolic mevalonate (MVA) and the plastic methyl-D-erythritol 4-phosphate (MEP) pathways in plant trichomes. It is noteworthy that the MEP pathway enzymes were more abundant in trichomes of *Artemisia. annua *[23], in which sesquiterpene metabolism dominates. These findings suggest that terpene metabolism in plant trichome is somehow different from the received theory that MVA pathway is predominantly responsible for the generation of sesquiterpenes, whereas MEP pathway is mainly for monoterpenes, diterpenes and tetraterpenes [24]. Unfortunately, due to the relatively limited sequences available in the EST library, analysis of the MVA and MEP pathways in tobacco trichomes seems extremely difficult, and will certainly be a focus of future analyses.

### Stress response

Trichomes provide the first line of plant defense against biotic and abiotic stress. Unsurprisingly, a lot of sequences in tobacco trichome EST library were identified as stress-related genes based on their homology with the known sequences from *Arabidopsis, Capsicum *and other plant species. PR-proteins specifically induced by pathological or related situation form the main system of the biotic response [25]. Two endochitinases, one belonging to group IV of tobacco PR protein [GenBank: BAF44533], the other homologous to *Capsicum *PR protein [EMBL: CAA55812], expressed at dramatically higher levels in trichomes than in leaves (62 fold and 45 fold, respectively). In addition, 022G07 [Swiss-Port: Q56S59] coding T-phylloplanin was enriched in trichomes by 22 fold. This surface-localized protein, synthesized only in the head cells of short glandular trichomes of tobacco, has provided a protein-based surface defense system against pathogens [26]. Abiotic stress responses probably form another important defense function of trichome. A cluster of genes related to osmotic, temperature, light, mechanical wounding, and oxidative were annotated in the trichome EST library. Genes responding to chemicals, such as toxin, nutrients and hormones, were also abundant in trichome EST library. Some of them expressed at even higher levels in trichomes than in leaves. 008E07 and 049E02, identified as heat responsive genes [GO: 0009498] had 4.2 fold and 6.4 fold higher levels in trichomes, respectively. 001E03, putatively responsive to ethylene stimulus [GO: 0009723], was expressed 17.4 fold higher in trichome. 009H02 [GenBank: AAG43549], responsive to abscisic acid, and 063C04 [EMBL: CAJ13709], responsive to auxin were both more actively expressed in trichomes. As for epidermal structures, trichome cells seem to be more sensitive to environment factors than leaf cells. Previous studies have indicated that nitrogen supply, water stress, mechanical wounding, light quality and intensity have significant effects on trichome development, metabolism, exudate content and chemical stability [27]. Our results provide some molecular proofs of the interaction between trichomes and the environment. However, reports on trichome development and metabolism affected by plant hormone are not available. A comprehensive understanding of the effects of hormone on trichomes will help to find new ways of the regulation of chemical compounds in the leaf surface.

## Conclusion

We analyzed gene expression in the leaf trichomes in *N. tabacum *using EST sequencing and cDNA microarray technologies. The overview of transcriptome of tobacco trichome was different from that of other plant species. Primary metabolism-related genes accounted for larger proportion in the EST library, while secondary metabolism and resistance-related genes were more highly expressed in trichomes than in leaves. Genes identified as involved in the terpene metabolism and stress response might be good starting points of further functional investigations. A more comprehensive understanding of transcriptome features, and the identification of genes involved in important functions should pave the way for more precise regulation of metabolic process in plant trichomes.

## Methods

### Plant material

Tobacco plants of *Nicotiana tabacum *L. cv. K326, a variety of excellent aroma quality, widely used for years in China, were cultivated in fields in Pingdingshan County, Henan province of China, according to the farming practice routinely used in the locality. Developmentally mature leaves (40~50 cm in length, 90d after transplantation) were collected for cytology examination, trichomes isolation and RNA extraction.

### Cytology examination

Tobacco leaves were cut into thin slices (< 2 mm) and the surface examined by fluorescence (BX51, Olympus) and scanning electron microscopy (S-3400N, Hitachi). For ultrastructure analysis, leaf slices were fixed overnight in a 4% solution of glutaraldehyde in 0.1 M phosphate buffer (pH 7.2) at 4°C, and post-fixed with 1% OsO_4 _in the same buffer for 1 h. The fixed tissue blocks were dehydrated in an alcohol series of 30, 50, 70, 90 and absolute ethanol, before being imbedded in epoxy resin. 69 nm sections were cut with a diamond knife of a LKB-NOVa ultramicrotome, stained with uranyl acetate-lead citrate, and observed in a JEM -1 00CX TEM operating at 80 KV.

### Trichome isolation and RNA extraction

Trichomes were isolated according to the cold-brushing method [28]. The leaves were frozen in liquid nitrogen and brushed on a slanting stainless steel board with a suitable hairbrush. The isolated trichomes and leaves-minus-trichomes (i.e. the leaves after brushing) were preserved in liquid nitrogen for total RNA extraction following the standard protocol of RNeasy Plant mini kit (Qiagen, Germany). Quality and quantity of RNA were assessed by formamide gel electrophoresis.

### Trichome EST library construction

The trichome cDNA library construction was done as previously described [29]. Briefly, trichome mRNA was isolated by 2 rounds of oligo-(dT)-cellulose column chromatography. cDNA synthesis from 2 μg of purified mRNA and library construction were carried out with a SMART™ cDNA Library Construction Kit according to the manufacturer's instructions.

A total of 5300 clones were subjected to single-pass sequencing reactions from the 5'-end with a model 373 sequencer (Applied Biosystems). Vectors and sequences < 400 bp or containing > 1.5% of imprecise nucleotides were removed. Sequences were edited manually to remove contaminants originating from the vector and poor quality 3'-sequences. Sequence comparisons against the GenBank non-redundant protein database were performed by using the BLASTX algorithm. A match was agreed when the E-score was > 120 (optimized similarity score), with 65% sequence identity over a minimum of 30 deduced amino acid residues. EST sequences were grouped, where appropriate, into sequence clusters by using TIGR ASSEMBLER. In addition, the sequences of each contig were aligned by using the fragment assembly program of the Wisconsin Sequence Analysis Package, and consensus sequences were generated with 90% identity for a minimum of 40 nucleotides.

### Microarray analysis

2381 ESTs from trichomes were selected, and the corresponding cDNA clones were amplified by PCR using T3 and T7 primers. After purification, the amplified cDNAs were spotted onto the glass microscope slides. Each cDNA clone was arrayed 3 times in random positions. RNA extracted from trichomes and leaves-minus-trichomes were reverse transcribed. cDNAs were labeled with succinimidyl ester Cy3/Cy5. The microarray, with samples of trichomes/leaves-minus-trichomes, was carried out in duplicate with the dyes reversed. The threshold ratio of detection was 2.0. A quality control procedure was conducted before data from the 6 replicates of 2 independent arrays were averaged. Finally, only spots that exhibited signals higher than those of the array backgrounds in both hybridizations and whose signals were 2 fold higher than the background of both hybridizations were further analyzed.

### RT-PCR analysis

RT-PCR analysis of selected genes was used the SuperScript One-Step RT-PCR System following the manufacture's protocol. 30 cycles of denaturation for 1 min at 94°C were followed by annealing for 2 min at 50-55°C and extension for 2 min at 72°C, followed by a final extension for 5 min. The primers sequence for each of the selected genes were: 002A01 (Forward 5' GACTT GCGAGGCAACAAGG 3', Reverse 5' GTGCTGCTTCATACAAACTC 3'), actin (Forward 5' TTGACGGAAAGAGGTTAT 3', Reverse 5' GTTGGAAGGTGCTGAGAG 3'), 054F03 (Forward 5' GACTTATGAAAGAGGGAGG 3', Reverse 5' AAGAGGTAGTGGAGGATG 3'), 004B10 (Forward 5' GCTATTGCCCAAGTTGTTTC 3', Reverse 5' GTAGCAGGCTATCTCGTT 3'), 001B03 (Forward 5' GCTGCCTCATTCCCTGTT 3', Reverse 5' GTTGGAAGGTGCTGAGAG 3'), 029B08 (Forward 5' AGGCAAATCCCAGACAGACC 3', Reverse 5' TAGCCAACATACCCATC 3'). Parallel reactions using actin primers were used to normalize the amount of template cDNA added in each reaction.

## Authors' contributions

HC contributed to the conception and design, interpretation of the data, drafting and revising the manuscript. SZ worked on array design, hybridization, as well as data analysis and submit. HY carried out bioinformatics analysis, especially EST assembly and annotation. HJ constructed the trichome cDNA library. XW was involved in data analysis and manuscript revision.

All authors read and approved the final manuscript.

## Supplementary Material

Additional file 1 **Sequence Information of 207 up Regulated ESTs in Tobacco Trichome**. The data represent all the 207 unigenes which expressed much higher in trichomes than in leaves. The experiment data have been submitted to ArrayExpress http://www.ebi.ac.uk/arrayexpress, with accession No. of E-MEXP-3148. Click here for file
